# ‘Better see a doctor?’ Status quo of symptom checker apps in Germany: A cross-sectional survey with a mixed-methods design (CHECK.APP)

**DOI:** 10.1177/20552076241231555

**Published:** 2024-02-29

**Authors:** Anna-Jasmin Wetzel, Roland Koch, Nadine Koch, Malte Klemmt, Regina Müller, Christine Preiser, Monika Rieger, Inka Rösel, Robert Ranisch, Hans-Jörg Ehni, Stefanie Joos

**Affiliations:** 1 Institute of General Practice and Interprofessional Care, University Hospital Tübingen, Tübingen, Germany; 2 Institute of Software Engineering, University of Stuttgart, Stuttgart, Germany; 3 Institute of Applied Social Science, University of Applied Science Würzburg-Schweinfurt, Wurzburg, Germany; 4 Institute of Philosophy, 9168 University of Bremen, Bremen, Germany; 5 Institute of Occupational and Social Medicine and Health Services Research, University Hospital Tübingen, Tübingen, Germany; 6 Institute of Clinical Epidemiology and Applied Biometry, University Hospital Tübingen, Tübingen, Germany; 7 Faculty of Health Sciences, 26583 University of Potsdam, Potsdam, Germany; 8 Institute of Ethics and History of Medicine, University Hospital Tübingen, Tübingen, Germany

**Keywords:** Symptom checker apps, primary care, self-triage, self-diagnosis, digital health, eHealth

## Abstract

**Background:**

Symptom checker apps (SCAs) offer symptom classification and low-threshold self-triage for laypeople. They are already in use despite their poor accuracy and concerns that they may negatively affect primary care. This study assesses the extent to which SCAs are used by medical laypeople in Germany and which software is most popular. We examined associations between satisfaction with the general practitioner (GP) and SCA use as well as the number of GP visits and SCA use. Furthermore, we assessed the reasons for intentional non-use.

**Methods:**

We conducted a survey comprising standardised and open-ended questions. Quantitative data were weighted, and open-ended responses were examined using thematic analysis.

**Results:**

This study included 850 participants. The SCA usage rate was 8%, and approximately 50% of SCA non-users were uninterested in trying SCAs. The most commonly used SCAs were NetDoktor and Ada. Surprisingly, SCAs were most frequently used in the age group of 51–55 years. No significant associations were found between SCA usage and satisfaction with the GP or the number of GP visits and SCA usage. Thematic analysis revealed skepticism regarding the results and recommendations of SCAs and discrepancies between users’ requirements and the features of apps.

**Conclusion:**

SCAs are still widely unknown in the German population and have been sparsely used so far. Many participants were not interested in trying SCAs, and we found no positive or negative associations of SCAs and primary care.

## Introduction

Although the internet as a source of health information is ranked the lowest considering trust by patients compared to other sources such as the social environment or doctors, it is still frequently consulted.^1^ Studies of health information seeking behaviour (HISB) indicate that 72% of Germans use the internet as a source of health information.^2^ Further studies identified general practitioners (GPs) as the primary source of HISB in Germany, followed by medical specialists and pharmacists.^3^

HISB is defined by the type and quantity of health-related information individuals seek, the particular strategies employed to acquire information, and the sources they rely on.^4^ This concept can be can be integrated into the broader framework of ‘access to information’, which is defined by the Universal Declaration of Human Rights as the entitlement ‘to actively seek, receive and share information and ideas through any means, without regard to geographic boundaries’. This underscores the importance of recognising access to health information within this framework.^5^

Symptom checker apps (SCAs) are one example of a newly developed type of health app that can be used as a source in the context of HISB. SCAs are tools designed for medical laypeople and promise to offer self-triage as well as the classification of symptoms in a low-threshold setting. Users enter symptoms and receive an assessment of their conditions with plausible diagnosis suggestions as well as recommendations for further actions, such as seeing a doctor, going to an emergency room or staying at home and waiting.

As the relevance of these tools may increase due to an increasing scarcity of resources in the health care system such as lack of a sufficient number of GPs, adequate handling is important. In several countries, SCAs are also offered by federal organizations, such as NHS111^6^ and the German National Association of Statutory Health Insurance.^7^ Considering the reported lack of accuracy, it is important that SCA recommendations and the expected benefits resulting from their usage are based on solid evidence. SCAs are often claimed to be inaccurate and described as risk-averse, that is, they recommend seeking health care in non-urgent scenarios.^8–11^ A recent study from 2022 based on the work of Semigran et al. (2015)^8^ examined whether the triage performance of SCAs has improved in the last five years, and found that, on average, it has not.^12^ In two SCA use cases, triage performance (advice on when emergency care is necessary and when no health care service is required) has decreased in the last 5 years.^12^ The authors stated that triage capability still varies widely depending on the chosen app and the specific question—for example, the overall diagnostic accuracy differed between 25% and 80%.^12^ A recent study found that SCAs recommended the use of primary care resources in 81% of the cases for which NICE (National Institute for Health and Care Excellence) clinical knowledge summaries indicate self-care.^9^ Moreover, the safety of action recommendations decreases with urgency, which may result in an undersupply.^9^ A recent study compared laypersons’ triage accuracy with the accuracy of SCAs and found that the latter was not superior.^13^ Despite the frequently insufficient accuracy^8–10,14^ SCAs satisfy their users.^15,16^ However, it remains unclear whether users are aware of their poor performance or if they continue to see the benefit in using SCAs despite the inaccuracy.

It is necessary to gain knowledge about SCA user groups, their recent extent of use and the consequences in the context of primary care. To date, the extent of SCA use and the reasons for their use are largely unknown.

To our knowledge, no previous studies have investigated the recent usage rate of SCAs or users’ willingness or reluctance to use SCAs in Germany. Our study is part of the multi-disciplinary joint project CHECK.APP that investigates the ethical, legal and social implications of SCAs in Germany.^17^ In our study, we conducted a survey to gain better knowledge about SCA use among the German population. Furthermore, we used user diaries and qualitative interviews to gain an in-depth understanding of user behaviour and potential implications for primary care. In this paper, we will present results from the survey and focus on the following research questions:

1. What is the degree of SCA utilization in Germany and which SCA software is the most used?
2. What role do SCAs play as part of the HISB?
3. Are there associations between SCA usage and the number of consultations of the GP and between usage and satisfaction with the GP present?
4. What are the reasons for the non-use of SCAs?

While questions 1–3 aim to provide a general overview about the usage of SCAs in Germany, the last research question aims to provide a more detailed understanding of the group of non-users, who have been hardly considered in empirical research.

## Methods

### Design

An explorative, cross-sectional survey was conducted using a mixed-methods design. Open-ended responses were assessed and processed using the qualitative data. The survey was available online and in a paper-and-pencil version.

### Measurements

SCA usage was assessed in three categories: ‘not an SCA user, and never heard of SCAs’, ‘not an SCA user, but have already heard of SCAs’ and ‘SCA user’. We defined subjects as ‘SCA users’ if they stated that they had used SCAs at least once. SCA users were asked to report the software that they had already used in filter questions. SCA non-users were asked if they were interested in trying SCAs. Open-ended responses were assessed to explore intentional non-use.

Subjects were asked about the number of GP consultations per year on a five-level scale (less than once a year, once a year, every 3 months, once a month, and more than once a month). HISB was assessed with multiple-choice selection (‘Assuming you have health complaints, where do you most commonly seek information?’ Friends, books, doctors, Internet and SCAs). Satisfaction with GPs was assessed using an adapted version of the Questionnaire for Satisfaction in Ambulatory Care,^18^ a validated four-item scale that assesses patient satisfaction with ambulatory care considering the concept of patient involvement. Answers on every item range between 0 and 3 and sum up to a score between 0 and 12. Permission to use the evaluated scales was requested individually via email and approved by the authors.

### Data collection

Data were collected from November 2020 to June 2021. Different recruitment channels were used to reach a wide variety of participants and ensure that enough participants who used SCAs had a sufficient number of cases for statistical analysis. First, 50,000 German citizens were contacted via an e-mail to participate in the survey. The intended recipients were selected by an external partner (T + R Dialog Marketing (Berlin, Germany) and Acxiom (Neu-Isenburg, Germany)). Further participants were selected using e-mail lists at the University of Tübingen and the University Hospital of Tübingen, social media, and by cooperating with GPs.

Inclusion criteria for participation in the survey were the ability to give consent and German language skills of at least the B1 level of the Common European Framework of References for Languages.^19^ The exclusion criterion was to have completed training as a medical doctor, as we assumed that the handling of SCAs by doctors differs from that of laypersons. In accordance with the ethical vote, all data were assessed anonymously, and participants provided written informed consent before starting the survey.

### Quantitative analysis

Data processing and statistical analyses were conducted using R Version 4.1.1^20^ and R Studio Version 1.4.^21^

#### Weighting

To maximise representativeness, data weighting based on summary statistics provided by the German Federal Statistical Office for the German population^22^ was used. A raking procedure (*anesrake* package^23^) was applied to weigh the participants according to age, school education and gender. All analyses used these weights to reduce the bias associated with frequency deviations in the German population.

For descriptive data, frequencies (*n*) and percentages (%) were generated for categorical variables, and means and standard deviations (SD) were calculated. The median and interquartile range (IQR) were generated for continuous variables. To compare the three SCA (non-)user groups, the Kruskal–Wallis rank-sum test for complex survey samples was conducted for nominal- or ordinal-scaled variables.^24^ To compare the HISB in different age groups with regard to SCA use, a weighted Pearson *χ*^2^ test was performed. Due to the small number of missing data in the dataset and the assumptions of missing completely at random (MCAR), pairwise deletion was used in the case of missing single variables. Analyses that examined the associations between GPs and SCA use involved participants who stated that they had a GP.

### Qualitative analysis

Data processing and qualitative analyses were conducted using MaxQDA Version 2020.^25^ Open-ended responses regarding reasons for intentional non-use were analyzed using inductive thematic analysis following the principles outlined by Braun and Clark (2014).^26^ The researchers had backgrounds in general practice (RK, MD, Dr, male), sociology (MK, Dr, male) and ethics (RM, Dr, female). Preliminary themes were discussed with members from other subprojects, and discrepancies that arose during theme synthesis were discussed with all authors and could be resolved by refining the themes. Data saturation was achieved in our study, as no further substantial aspects or new themes emerged during the data collection process. All authors approved the final themes.

### Data integration

The quantitative and qualitative data were structured based on the thematic focus of the discussion section. This allowed merging^27^ and higher-order integration of both quantitative and qualitative results were based on interpretations, which are described in the Discussion section.

## Results

### Respondents and sociodemographic characteristics

A total of 871 participants (*n* = 116, paper–pencil; *n* = 755 online) completed the survey. Of these, two had missing 5,6 values on SCA usage, and 19 were physicians and were therefore excluded; finally, 850 subjects were included in the analysis.

The sociodemographic characteristics of the participants are presented in Table 1. The three groups differed significantly in terms of age (*p* = .01) and educational level (*p* = .03). No group differences were observed with regard to gender, living area and chronic conditions, and being affiliated with a GP.

**Table 1.** Sociodemographic characteristics of the three user groups.

Characteristics	Not An SCA User and Never Heard of SCAs, *N* = 573^a^	Not An SCA User, But Already Heard of SCAs, *N* = 213^a^	SCA User, *N* = 64^a^	*P*-value^b^
**Age**	52 (17)	48 (15)	42 (14)	.008
**School education**				.027
No graduation degree	10 (1.8%)	0 (0%)	0 (0%)	
Graduation after 9 years	198 (35%)	30 (14%)	10 (16%)	
Graduation after 10 years	173 (31%)	77 (36%)	28 (45%)	
A-levels	180 (32%)	106 (50%)	24 (39%)	
**Gender**				.2
Female	306 (54%)	93 (44%)	28 (45%)	
Male	258 (46%)	120 (56%)	35 (55%)	
**Area**				.4
Major city	47 (8.4%)	25 (12%)	4 (6.9%)	
Suburbs of a major city	31 (5.5%)	18 (8.3%)	5 (8.1%)	
Midsized city – town	327 (58%)	130 (61%)	31 (49%)	
Village	157 (28%)	40 (19%)	23 (36%)	
**Chronic condition**				.3
No	290 (52%)	102 (49%)	40 (65%)	
Yes	234 (42%)	99 (48%)	22 (35%)	
Prefer not to say	31 (5.6%)	7 (3.5%)	0 (0%)	
**Personal GP**	528 (94%)	193 (91%)	61 (94%)	.4

GP: general practitioner; SCA: symptom checker app.

^a^ Mean (SD); *n* (%).

^b^ Wilcoxon rank-sum test for complex survey samples; chi-squared test with Rao & Scott's second-order correction.

### What is the degree of utilization of SCAs in Germany? Which SCA software is most used?

**Figure 1.**
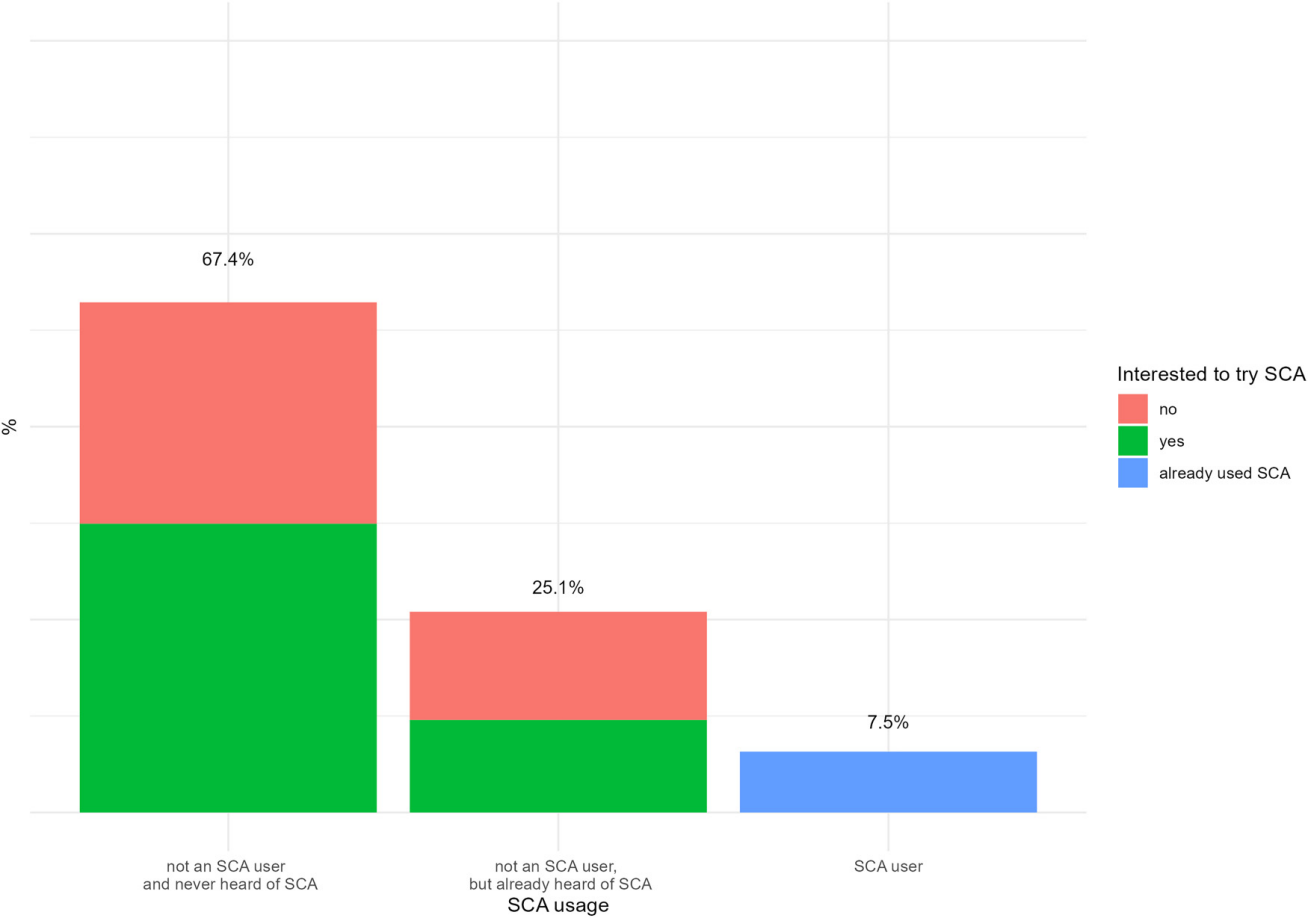
SCA usage and interest in trying SCAs. SCA: symptom checker app.

The SCA user group comprised 64 (7.5%) participants; the group of non-users who had already heard of SCAs comprised 213 (25.1%) participants, and non-users who had never heard of SCAs before were 573 (67.4%). Of the two non-user groups, 46% (*n* = 362) stated that they were interested in trying SCAs. Figure 1 shows the rate of usage stratified by interest in trying SCAs. Table 2 shows the most frequently used SCAs.

**Table 2.** Most used SCAs.

Most Used SCAs	*N* = 64^a^
NetDoktor SC	29 (45%)
Ada	20 (30%)
Other SCAs	19 (30%)
Diagnose Medizin app	3 (4.3%)
Symptomate	3 (4.3%)
WebMD	2 (2.9%)

SCA: symptom checker app.

^a^ 1n (%).

### Which place do SCAs take as part of HISB?

**Figure 2.**
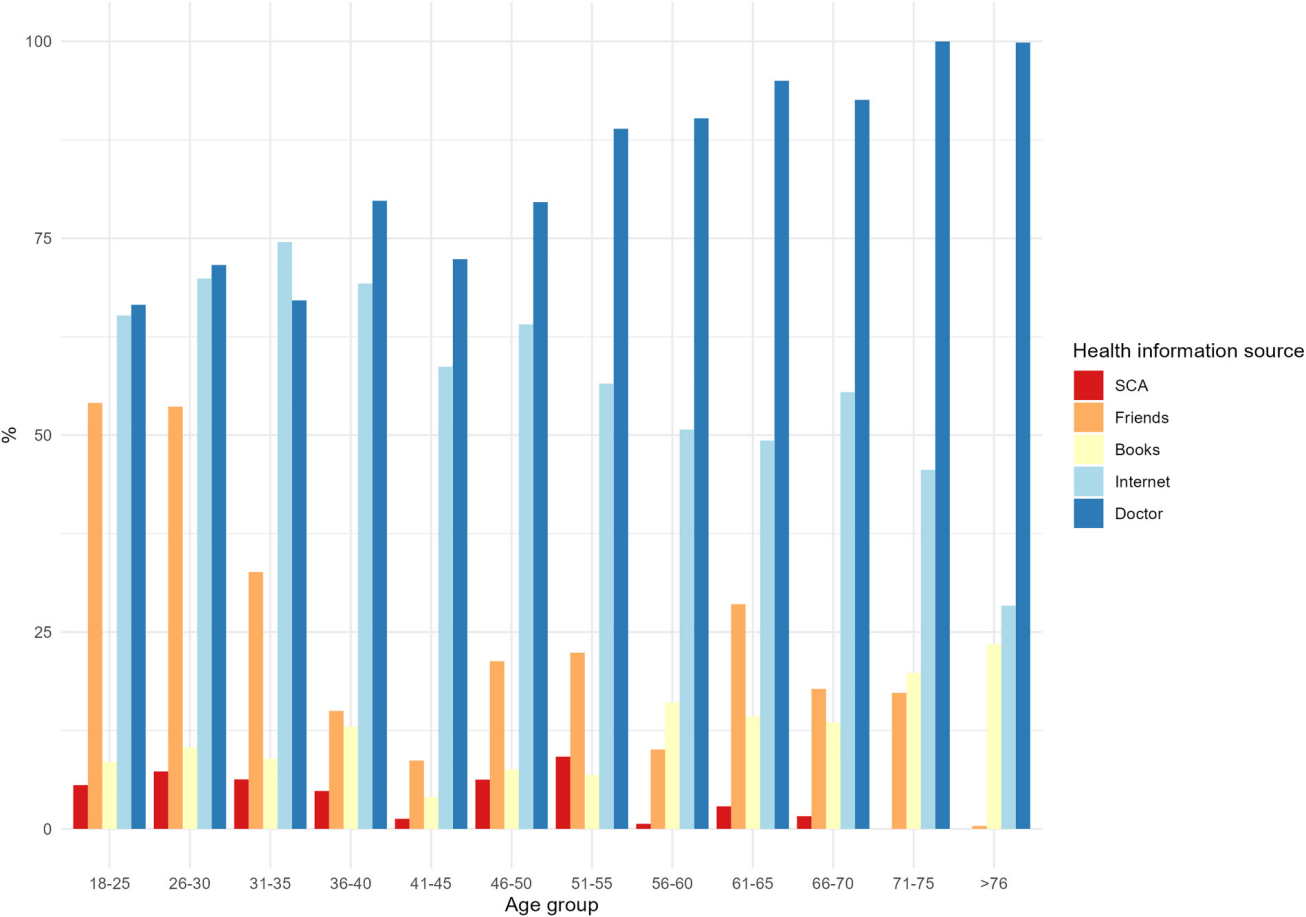
HISB stratified for the age group. HISB: health information seeking behaviour.

The analysis of HISB stratified by age revealed significant group differences, with *χ*^2^(44) = 138.06, *p* < .001. Younger participants (18–30 years old) informed themselves more frequently via friends and less frequently via a doctor than those over 35 years of age. People over the age of 46 used books more frequently to inform themselves. SCAs were most frequently used by participants aged 51–55 years (Figure 2).

**Figure 3.**
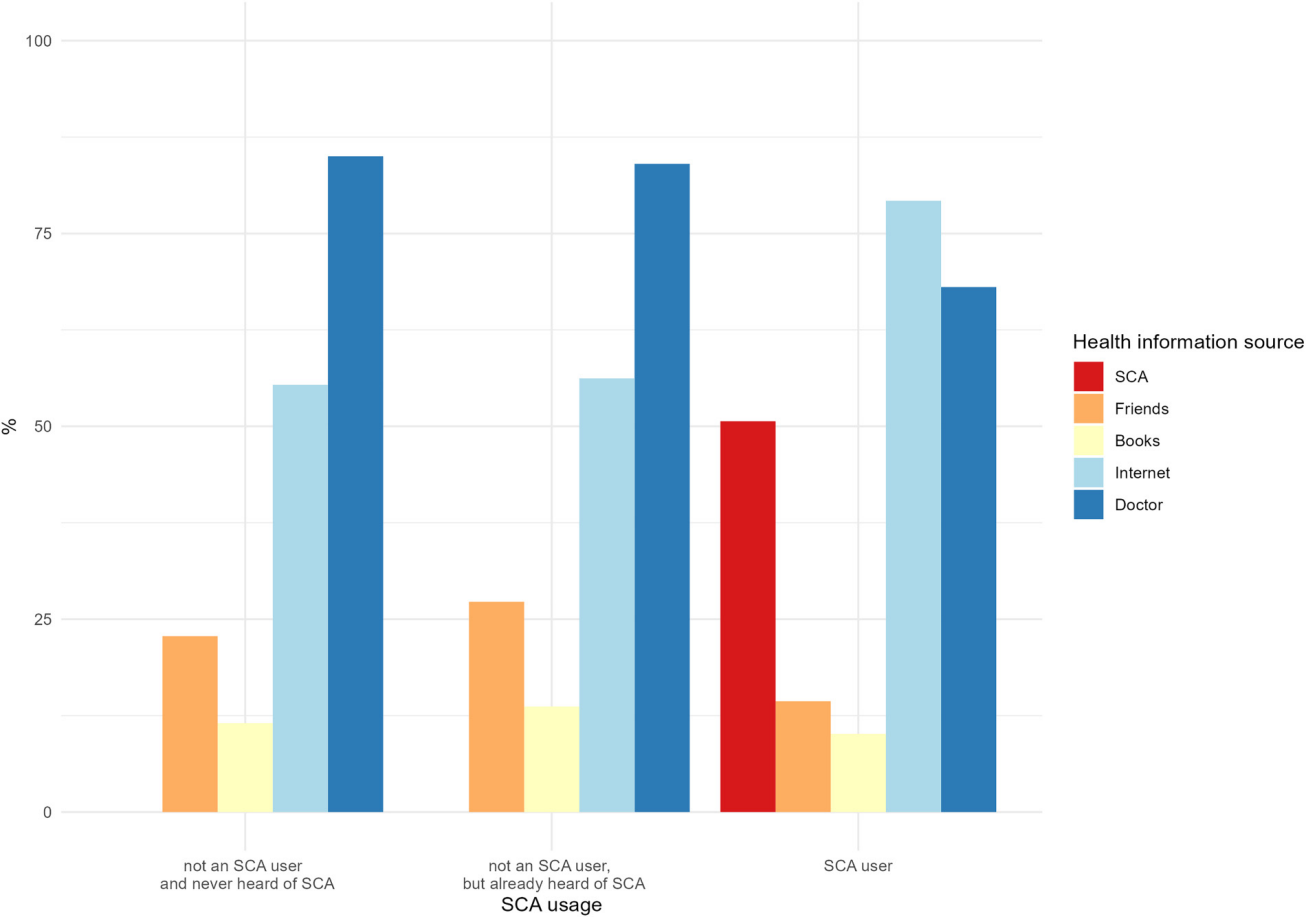
SCA use stratified for HISB. HISB: health information seeking behaviour; SCA: symptom checker app.

We regarded HISB as stratified for SCA user groups, and the proportions of health-seeking behaviour differed for the (non)-user groups of SCAs, with *χ*^2^(8) = 355.12, *p* < .001. One can observe that the proportion of participants who informed themselves via a doctor, friends and books was smaller in the SCA user group than in the other two groups. SCA users also consulted the Internet more frequently than both the non-user groups (Figure 3).

### Are there associations between SCA usage and the number of consultations with the GP or the satisfaction with the GP?

Group differences in SCA usage and satisfaction with the GP were investigated and showed no significant differences, with *χ*^2^(2) = 0.59, *p* = .74*.* Moreover, no group differences were observed between SCA usage and the number of reported GP consultations and SCA usage, with χ^2^(2) = 2.95, *p =* .23 (Table 3).

**Table 3.** Variables considering GP care stratified for SCA use.

Characteristics	Not An SCA User and Never Heard of SCAs, *N* = 573^a^	Not An SCA User, But Already Heard of SCAs, *N* = 213^a^	SCA user, *N* = 64^a^	*P*-value^b^
ZAPA	10.00 (8.00, 12.00)	10.00 (8.00, 12.00)	10.00 (8.00, 11.00)	0.7
Number of GP visits				0.2
Less than once a year	45 (8.4%)	17 (8.9%)	1 (1.2%)	
Once a year	98 (18%)	61 (32%)	14 (23%)	
Once half a year	153 (29%)	45 (23%)	23 (37%)	
Every three months	178 (33%)	51 (26%)	22 (37%)	
Once a month	42 (7.8%)	11 (5.5%)	1 (1.4%)	
More than once a month	20 (3.7%)	8 (4.0%)	0 (0%)	

GP: general practitioner; SCA: symptom checker app.

^a^ Median (IQR); *n* (%).

^b^ Kruskal–Wallis rank-sum test for the complex survey samples.

### Reasons for the intentional non-use of SCAs

**Figure 4.**
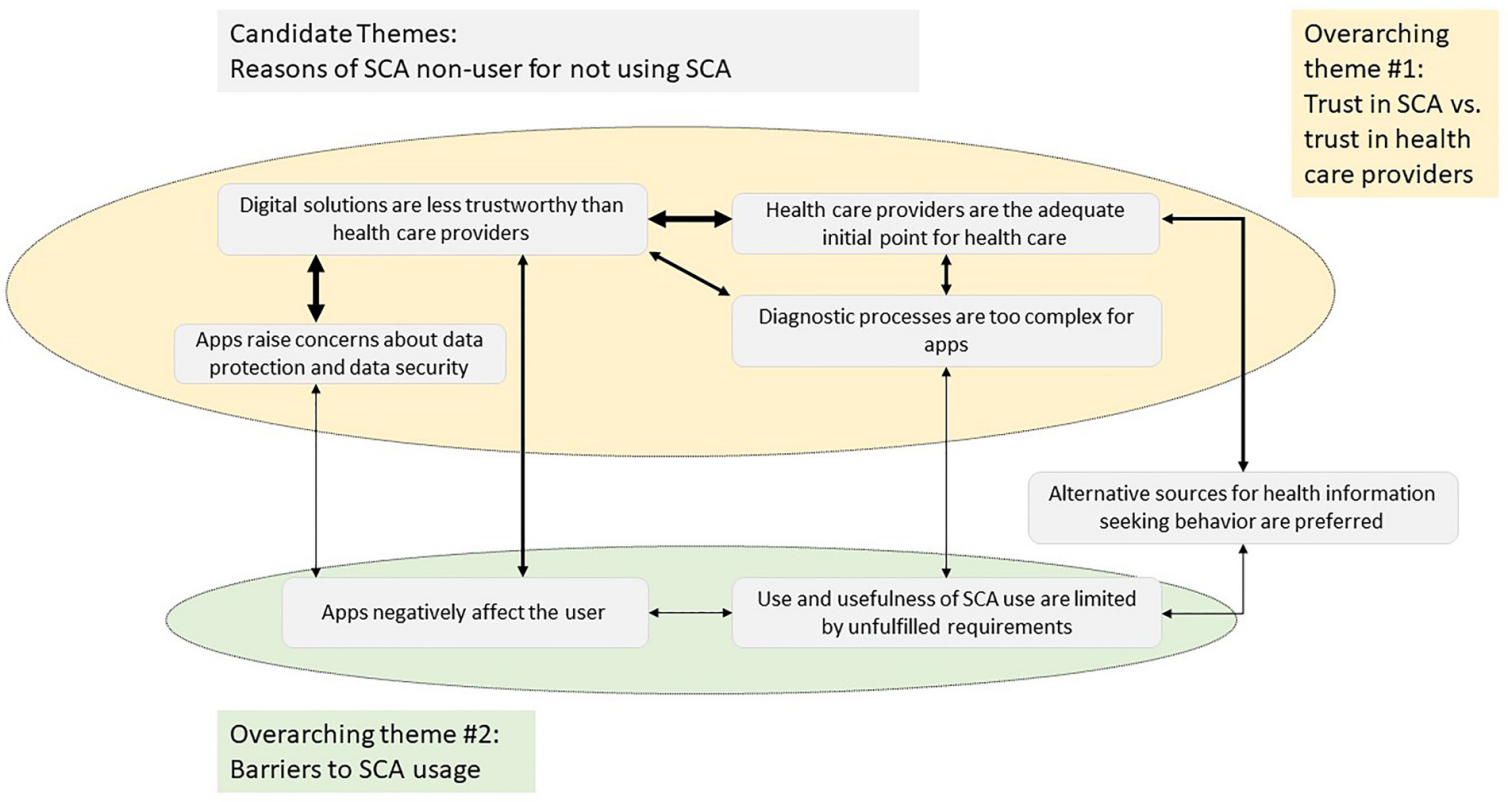
Themes of the thematical analysis considering the open-ended response to reasons for the intentional non-use of SCAs. SCA: symptom checker app.

Intentional non-users of SCA were asked, through an open-ended question, whether they were interested in trying SCAs, and if not, they were requested to give their reasons. We were able to generate several relevant and complex themes through a data analysis. The reflexive thematic analysis of the reasons for non-use yielded two overarching themes (Figure 4).

#### Trust in SCA vs. trust in health care providers

The first overarching theme comprised a comparison of trust in SCAs with trust in healthcare providers. In general, non-users expressed concerns about the trustworthiness of SCAs; in contrast, trust in the medical competence of established healthcare providers was higher. They indicated that healthcare providers are more capable of asking the ‘right’ questions about symptoms and consequently of making a correct diagnosis. They also preferred health care providers, who were perceived as an adequate initial point for health care and were able to offer physical examination as well as the opportunity to discuss findings. In addition, the trustworthiness of health care providers in ethical terms was also given more weight. Privacy and security concerns were expressed, which also influenced the perception of SCA's trustworthiness. From the perspective of non-users, SCAs are ethically less trustworthy than health care providers. Due to high trust in the established health sector, both in terms of medical and ethical competence, SCAs were assessed as irrelevant by non-users.

*‘I have more trust in the doctor than in an algorithm’ (327)*

#### Barriers to SCA usage

The second overarching theme comprised the potential impact and causes of a discrepancy in the requirements of the user and the app. SCA apps were described as ‘not *for everyone*’. Non-users saw themselves as unsuitable for SCA use due to their age, history, health conditions, lack of interest, lack of access to devices or lack of health literacy. Concerns were also expressed about the potential impact of SCA use, ranging from illness anxiety and fear of hypochondriasis to addiction.

*I am a bit of a hypochondriac and I fear that if I used such devices I would think I was ill even more often than I do now. (240)*

On another theme, non-users indicated that they preferred other ways of seeking information on health topics:

*I get enough information from friends and family about possible health problems. (178)*

## Discussion

In our study, SCAs still seemed to play a minor role in HISB and primary care in Germany, with a usage rate of 7.5%. The most commonly used SCA was the NetDoktor Symptom-Checker (45%), followed by Ada (30%). Surprisingly, the age group of 51–55 years reported the most frequent use of SCAs. As expected, SCA users informed themselves less frequently through doctors, books and friends.

### Distribution and classification of SCAs in HISB

Our results considering the Internet research in the context of HISB were in line with other findings from Germany^1–3^ and showed that the Internet research is the most used source in the context of HISB in addition to doctors’ appointments. However, SCAs seem to play a minor role in HISB, as they had the lowest usage rate compared to the other assessed sources. The popularity and usage of SCAs in Germany are still subordinate to the use of health apps in general. In a German federal innovation analysis conducted in 2018, 54% of the participants stated that they were using health apps,^28^ and the usage rate of these apps in general is consequently considerably higher than the low usage rate of SCAs.

We expected to observe a usage peak of SCA in the younger age group, as suggested by Meyer et al.;^29^ however, the age group that reported the highest SCA usage rate in our data was middle adulthood. One reason for the lower usage rate in younger age groups may be the preference for using general internet research as a competitor of SCAs. Further investigations are necessary to exclude the possibility of a sampling bias as a potential explanation for this finding.

Furthermore, we did not see older adults over 70 years of age using SCAs and only a minority of adults older than 60 years used SCAs. A study from 2018 reported that 16.5% of older adults in Germany used health apps and 37.5% used apps in general.^30^ The lack of SCA utilization among certain demographics, such as individuals aged 55 and above, increases the likelihood of health inequality if the importance of SCAs continues to grow.

A further important aspect to consider in future research is whether different HISB sources exert an influence on one another, considering access to health information and/or access to health care in general. It might be particularly interesting to investigate SCA use in diverse health-care systems where access to primary care varies significantly.

Our study was carried out during peaks of the COVID-19 pandemic. Self-diagnosis is an important element in pandemic control, so it is likely that the general use of symptom checkers might increase during times of pandemic.^31^ Various COVID-19 related symptom checkers had been developed to help citizens to self-diagnose a potential COVID-19 infection,^32^ e.g., in Denmark, where a SCA was developed within days and was used 150,000 times in a few weeks.^33^ In Germany, however, COVID-19 symptom checkers were not part of public pandemic management.

### Associations between SCA use and GP care

The majority (75%) of SCA use comprised applications that aimed to process symptoms toward a probable diagnosis (such as NetDoktor Symptom-Checker and Ada), indicating a potential demand for SCAs as a tool in general practice.^11,34,35^ A study from Switzerland in 2020 reported that out of 1000 participants, 546 had one or more health complaints within the previous two months – the majority of which were mainly dealt with by general practice. These results show the substantial strain that lies in the primary care health sector, which might be influenced by SCAs. However, the results of the present study indicate neither an association between SCA usage and GP consultations, nor an association between satisfaction with GPs and SCA use. The satisfaction rate with GPs in general was high in our sample, as it is usually for Germany^36^ and Europe.^37^ The association of satisfaction with GPs and SCA use may differ from that observed in our sample of other health systems with lower satisfaction rates.

In their study on the accuracy of SCAs, Ceney et al. (2021) concluded that SCAs may trigger additional resource utilization as it recommends, in over half of the cases, seeking health care, with primary care resources being suggested in approximately 81% of conditions where NICE clinical knowledge summaries indicate self-care.^9^ A Danish study investigating the utilization of health data through new technologies discovered that, alongside data from wearables, the results obtained from SCAs are among the most mentioned for GPs during medical consultations for interpretation.^38^ These findings may indicate an increasing relevance of interpreting symptom checker results in the context of primary care and are inconsistent with the promise of reduced health care utilization. Further studies have indicated risk-averse self-triage in the context of SCAs, in the sense that even rare diseases are not overlooked,^8,10^ decreasing the accuracy of the action recommendations in non-urgent scenarios.^9^ Schmieding et al. (2021) found that SCAs and laypersons performed the poorest in self-triage in non-urgency scenarios^13^; in contrast, another recent study reported a decrease in perceived urgency among users of the intended level of care after using a specific SCA.^39^

However, it remains unclear how laypersons handle especially non-urgent scenarios when using SCAs after receiving recommendations for action that suggest health care utilization. To prevent unnecessary utilization by SCAs, it is necessary to understand the relationship between primary care utilization, handling of prior knowledge, action recommendations of SCAs and decision-making in laypersons. A study from 2022 that examined the impact of SCA explanations on laypersons’ trust found that SCAs must tailor answers to each user's specific question and take the prior knowledge of the user into account.^40^ When laypersons had prior knowledge about a disease, the answers given by SCA had little influence on their trust. However, these answers had a significant influence on trust when the need for information was higher.^41^

### Reasons for intentional non-use of SCAs

One reason for the low usage rate was the lack of knowledge regarding the existence of SCAs (67.4%). In our sample, 25.1% stated that they knew about SCAs, but did not use it. Considering the non-user group that had already heard of SCAs, but had never used it, 47% expressed their disinterest in using SCAs. A qualitative analysis of the open-ended responses contemplating reasons for intentional non-use provided possible explanations. The general trustworthiness of SCAs was doubted, and it was perceived as an inadequate initial point for health care and unable to handle the complexity of diagnostic processes. Another reason was the perceived barriers to SCA use and limited benefits. Our results concerning privacy and security concerns align with other findings related to health apps in general.^42^ Moreover, our findings regarding the usefulness of SCAs also corroborate the results from the literature, which reported a lack of perceived usefulness as hindering mobile app use in general.^43^ The same study also reported a lack of app literacy as a further hindering factor, which coincides with our findings that suggest a mismatch of requirements between the user and SCAs.^43^

### Strengths and limitations

The strengths of this study include the mixed-method and interdisciplinary team approaches; therefore, different perspectives and methods were integrated to make a broad spectrum of studies possible.

However, our results may not be applicable across countries, especially where healthcare systems, primary care provision, and cultural and social conditions differ. The challenge of a heterogeneous wording about SCAs has previously been addressed and could not be solved completely in this study, too. As a consequence, we cannot exclude that some participants were unable to differentiate between a general Internet search and the use of SCAs, such as those accessed through a web browser. Furthermore, our data collection led to sampling bias toward younger and better-educated participants, we addressed this by weighting the data.

## Conclusions

SCA use is still in its early stages. We observed a sample that was divided into skeptics and potential users, SCAs may have an important impact; however, our study does not indicate whether this impact is positive or negative. This is also a chance for regulations to prevent negative consequences and promote the benefits of these applications. Contrary to our expectations regarding the advertisement of SCAs, based on our study, SCAs are still widely unknown in the German population and sparsely used. Further research that examines primary care utilization in the context of SCA usage by taking into account a system perspective is necessary to identify the added value and possible unnecessary utilization or misallocation of resources through SCAs.
